# High morbidity and mortality associated with primary bloodstream infections among pediatric patients with cancer at a Guatemalan tertiary referral hospital

**DOI:** 10.3389/fpubh.2022.1007769

**Published:** 2022-11-17

**Authors:** Sheena Mukkada, Mario Melgar, Craig Bullington, Alicia Chang, Maysam R. Homsi, Miriam L. Gonzalez, Federico Antillon, Yin Su, Li Tang, Miguela A. Caniza

**Affiliations:** ^1^Department of Global Pediatric Medicine, St. Jude Children's Research Hospital, Memphis, TN, United States; ^2^Department of Infectious Diseases, St. Jude Children's Research Hospital, Memphis, TN, United States; ^3^Unidad Nacional de Oncología Pediátrica, Guatemala, Guatemala; ^4^Hospital Roosevelt, Guatemala, Guatemala; ^5^University of Tennessee Health Sciences Center, Memphis, TN, United States; ^6^School of Medicine, Francisco Marroquin University, Guatemala, Guatemala; ^7^Department of Biostatistics, St. Jude Children's Research Hospital, Memphis, TN, United States

**Keywords:** bloodstream infection (BSI), bacteremia, pediatric oncology, supportive care, Guatemala

## Abstract

Infectious complications remain major contributors to adverse outcomes in patients treated for non-communicable disease, particularly in resource limited settings. We performed a 5-year retrospective study of primary bloodstream infections at a dedicated pediatric oncology center in Guatemala. Two hundred and twelve episodes occurring in 194 unique patients qualified for inclusion. Patients required intensive care unit admission in 55% of episodes and death occurred in 24% of episodes. Despite subspecialty support in infectious diseases, poor outcomes, including prolonged hospitalization and mortality, were frequent. Our findings suggest that investments in laboratory and clinical data collection are critical to understanding the contributors to poor outcomes and therefore to improving the quality of bloodstream infection management in resource limited settings.

## Introduction

While non-communicable disease has become a leading focus globally, infection remains a leading cause of morbidity and mortality in pediatric patients with cancer (1). This burden may be exaggerated in low-resource settings because of difficulty accessing care and insufficient diagnostic and treatment resources (2). Bloodstream infection (BSI) is the most frequent microbiologically proven infection in pediatric oncology patients presenting with febrile neutropenia. Beyond the effect on morbidity, BSI has been associated with increased length of hospitalization and resource consumption (3, 4). As blood cultures are the diagnostic tests performed most frequently in the workup of suspected infection, BSIs are index conditions for studying infection management and outcomes. We chose to examine BSI at a pediatric oncology referral center during a 5-year period when supportive care quality improvement was a focus. By reviewing data from a decade ago at this site, we identify challenges that middle to low resource centers across the globe should anticipate as they begin to expand services to deliver cancer-directed therapy.

## Methods

This is a retrospective study of all patients diagnosed with primary BSI while undergoing cancer-directed therapy at the Unidad Nacional de Oncología Pediátrica (UNOP), or the National Pediatric Oncology Unit, in Guatemala City, Guatemala, between January 1, 2011, and December 31, 2015. The study was approved by the Universidad Francisco Marroquin Ethics Committee and St. Jude Children's Research Hospital's Institutional Review Board.

UNOP is a 60-bed national referral center dedicated to managing malignant diseases in pediatric patients (aged 0–17 years). It reports an average of 500 new cancer diagnoses annually. Data were abstracted from the Pediatric Oncology Network Database and from the onsite infection surveillance and risk factors database. Missing data were extracted from patient charts, and positive blood cultures were cross-checked against the laboratory database. Information obtained included patient demographics, primary malignancy, treatment phase, extent and duration of neutropenia, infectious diagnoses, venous access status, and patient outcomes. The Centers for Disease Control and Prevention/National Healthcare Safety Network (NHSN) 2016 criteria were used to define primary BSI (5). Blood culture and identification/susceptibility testing, respectively, were performed by using BacTec and Vitek, applying CLSI guidelines. NHSN 2016 criteria were used to define central line–associated BSI (CLABSI), mucosal barrier injury (MBI) infection, healthcare-associated infection (HAI), and infection window (5). We used the NHSN criteria to define common commensal organisms (5). Given the difficulty of proving the pathogenicity of these organisms, they were documented as BSI only if they were recovered from more than one culture. Death and intensive care unit (ICU) admission were defined as BSI-related if they were documented as such and occurred within 30 days of organism isolation from the bloodstream.

Data were tabulated by using Epi Info^TM^ and analyzed by using SAS software (version 9.4). Population characteristics were illustrated *via* descriptive statistics. Associations of selected categorical variables were assessed *via* chi-square tests. Logistic regression was used to identify categorical predictors of selected outcome variables, and covariates with statistically significant effects (*P* < 0.05) were tested in multivariate models by step-wise selection.

## Results

**Figure 1:**
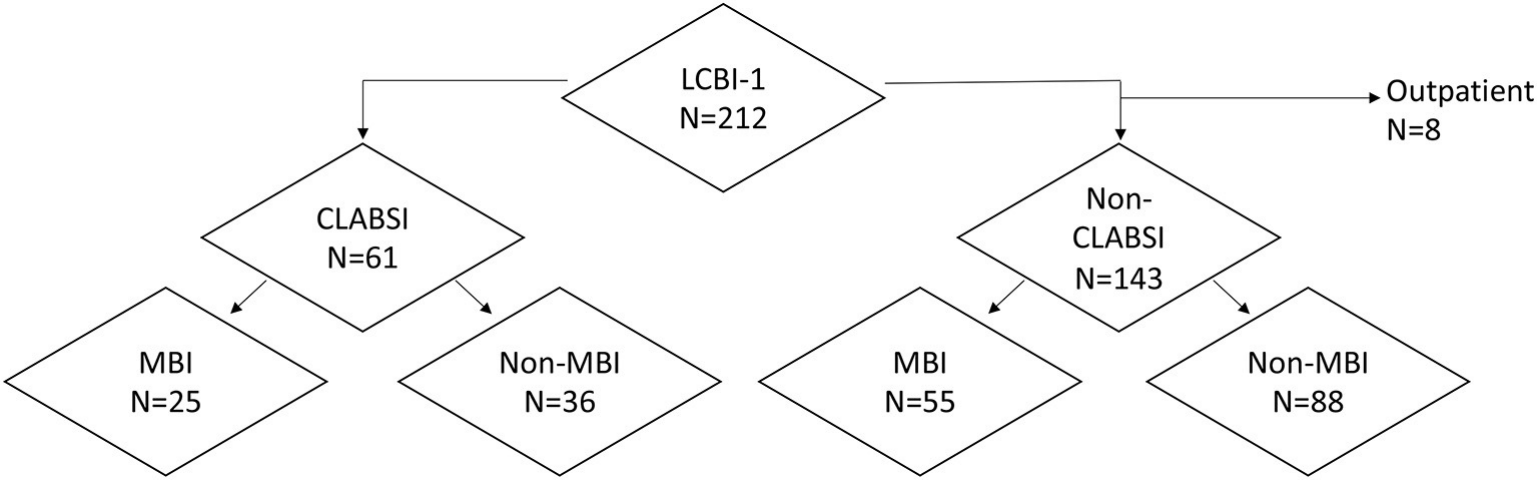
Distribution of bloodstream infections by Centers for Disease Control and Prevention/National Healthcare Safety Network (NHSN) 2016 surveillance classification type. CLABSI, central line–associated bloodstream infection; LCBI, laboratory-confirmed bloodstream infection; MBI, mucosal barrier injury infection.

Table 1 details the 212 episodes of laboratory-confirmed bloodstream infection (LCBI) in 194 unique patients, most of whom (80%) had hematologic malignancies. Seventy blood cultures were positive for common commensals. These were excluded from analysis due to pathogenicity not being verified by growth in a second blood culture. Ninety-six percentage of episodes occurred in patients who were hospitalized at the time of BSI; 77% of those were categorized as HAI. Among LCBIs, 37% met criteria for MBI, and 29% were categorized as CLABSIs (Table 1; Figure 1). Forty-one percentage of CLABSIs met criteria for MBI-LCBI. Both profound (ANC < 100 cells/mm^3^) and prolonged (>10 days) neutropenia were frequent in patients who developed BSI. Patients had a median duration of neutropenia of 16 days (range, 0–59 days) (Table 1). Median minimum ANC was 10 (range, 0–27160) during the BSI infection window. Morbidity and mortality in patients experiencing BSI were high; over half of all episodes required ICU admission, and 23% of episodes resulted in death.

**Table 1:** Characteristics of patients and bloodstream infections in study population.

**Patient demographics (*N* = 194)**	***n* (%)**	
Sex, male	109 (56.19)	
Median age, years (range)	9.53 (0.13–21)	
Primary disease = hematologic malignancy	155 (79.90)	
Treatment phase^a^, Induction	110	
Treatment phase^a^, Consolidation	30	
Number of patients contributing a single BSI	176 (90.72)	
Death during episode	48 (24.74)	
Death attributed to BSI	44 (22.68)	
**BSI characteristics (*****N*** **=** **212)**	***n*** **(%)**	
**Infection subtypes^b^**	
Healthcare-associated infection	160 (78.43)	
MBI	80 (37.74)	
MBI-LCBI	25 (11.79)	
Non–MBI-CLABSI	36 (28.77)	
**Infection predictors^b^**	
Neutropenia (< 500 cells/mm^3^)	162 (76.42)	
Profound (< 100 cells/mm^3^)	147 (69.34)	
Prolonged (>10 days)	101 (47.64)	
Mucositis	30 (14.15)	
**Venous access**	
Permanent line	16 (7.55)	
Peripheral line only	110 (51.89)	
Temporary line only	84 (39.62)	
No line	2 (0.94)	
**Infection outcomes**	
ICU admission	116 (54.72)	
ICU admission due to BSI	73 (34.43)	
Single pathogen isolation	193 (91.04)	
Median length of ICU stay, days (range)	7(1-376)	
Median length of hospital stay, days (range)	29.5 (0–479)	
**Multivariate analysis of clinically significant outcomes**	**OR (95% CI)**	***P***
Prolonged neutropenia and ICU admission	3.063 (1.595–5.885)	0.0008
Mucositis and ICU admission	3.366 (1.229–9.221)	0.0182
MBI and first ICU admission related to BSI	2.727 (1.453–5.120)	0.0018
Profound neutropenia and mortality	2.508 (1.082–5.816)	0.0321
Profound neutropenia and BSI-related mortality	3.177 (1.272–7.933)	0.0133

a Treatment phase refers to hematologic malignancies only.

b Infection subtypes and predictors are summarized individually so there are overlaps between classifications and *n* > 212.

BSI, bloodstream infection; CLABSI, central line–associated bloodstream infection; HAI, healthcare-associated infection; ICU, intensive care unit; MBI, mucosal barrier injury infection; MBI-LCBI, mucosal barrier injury–laboratory confirmed bloodstream infection.

BSI contributed significantly to poor outcomes observed in these episodes. Sixty-three percentage of ICU admissions were BSI-related. Univariate analysis demonstrated these admissions were more frequent in patients with prolonged neutropenia (*P* = 0.029), profound neutropenia (*P* = 0.003), or MBI (*P* = 0.004). MBI remained significantly associated with ICU admission due to BSI in the multivariate logistic regression model (OR 2.73, 95% CI 1.45–5.12) (Table 1). The only additional variable which retained significance in the multivariate analysis of BSI-related ICU admission was venous access status of having a temporary central venous catheter alone (Table 1). Additionally, 92% of deaths during BSI episodes were attributed to BSI. BSI-related mortality occurred more frequently in children with profound neutropenia (*P* = 0.0142) but was not associated with prolonged neutropenia. Cochrane-Armitage testing demonstrated no time trend in BSI-related mortality (*p* = 0.6207) despite contemporary implementation of an institution-wide pediatric early warning score intervention that decreased time to recognition of clinical deterioration events (6).

Of the 231 organisms isolated, 84% were Gram-negative bacteria. The most frequently isolated organisms were *Klebsiella pneumoniae* (*n* = 53), *Escherichia coli* (*n* = 38), and *Pseudomonas aeruginosa* (*n* = 23) (Table 2). Gram-positive infections were infrequent but occurred more frequently in patients with central lines (*P* = 0.01) (Table 2). Half of all patients had only a peripheral line at the time of BSI; however, catheter removal was associated with identifying a CLABSI (*P* < 0.0001). The CLABSI rate declined during the study period from 7.77 CLASBIs per 1,000 inpatient central venous catheter days to 2.44 CLABSIs per 1,000 catheter days in 2015.

**Table 2:** Pathogens isolated from bloodstream infections (*N* = 231).

**Pathogen**	***n* (%)**
*Klebsiella pneumoniae*	53 (22.94)
*Escherichia coli*	38 (16.45)
*Pseudomonas aeruginosa*	23 (9.96)
*Staphylococcus aureus*	18 (7.79)
*Acinetobacter baumannii*	17 (7.36)
Other gram negative bacteria	13 (5.63)
*Burkholderia cepacia*	12 (5.19)
Other Gram-positive bacteria	11 (4.76)
*Enterococcus faecalis*	8 (3.46)
Other Enterobacteriaceae	8 (3.46)
*Enterobacter cloacae*	7 (3.03)
*Pseudomonas* sp. other than *P. aeruginosa*	7 (3.03)
*Sphingomonas paucimobilis*	7 (3.03)
*Stenotrophomonas maltophilia*	6 (2.6)
*Candida* sp.	3 (1.30)

## Discussion

Our 5-year review of 212 LCBI episodes in a Guatemalan referral center reveals high morbidity and mortality in pediatric oncology patients who develop BSIs. This result contrasts with BSI-related mortality in pediatric oncology patients in high-income settings. The mortality rate was 1.8% in a contemporary multicenter European study and 11% in a 1995 multicenter Italian study (7, 8). A Turkish study of BSI in childhood cancer in the same era as ours (2010–2015) had a 7 day mortality of 2.7% (9). In addition to higher mortality, our study also revealed high resource consumption by patients with BSI. Hospitalization duration, a median of 29.5 days in our study, was only 19 days for an analogous patient population in Mexico City (4). The reasons for these disparities are not clear but are likely multifactorial, reflecting a combination of host and pathogen characteristics, and supportive care limitations. The low median ANC in this cohort suggests that this subpopulation is at high risk for infection, bleeding or other complications related to active disease and intensive chemotherapy. This may lead to other complications requiring inpatient stay. ANC was not reported in the study from Mexico, but median ANC from the Turkish study was 100 cells/mm^3^ (4, 9). The higher morbidity and mortality relative to the Turkish study suggests that other factors may have affected our outcomes.

Personnel and budget challenges often contribute to inconsistency in medical attention and management. While supportive care was protocolized through the locally developed algorithm for care escalation, the pediatric early warning score intervention at UNOP did not address specific points in infection management (6). During the time of the study, infectious disease specialists were employed by UNOP but even though antimicrobial treatment was standardized, the overall management of fever and neutropenia was not. One blood culture was performed in all patients with febrile neutropenia, but starting in 2015 all patients with a central line had both a central and a peripheral blood culture taken. In addition, the team started a Safe Vascular Access program in 2010, which included an insertion check list for peripheral and central lines and an insertion and care bundle. Policies and procedures for central venous catheter placement and management were part of institutional regulations, but these may have been applied inconsistently.

Our data suggests that improving infection control practices may have some impact on clinical outcomes, since non-MBI CLABSIs comprised 59% of CLABSIs. Evidence shows that use of standard catheter bundles may prevent some of these BSIs, even in an ICU environment (10). However, alternative approaches will be required to combat the MBI LCBIs, which were associated with the most severe outcomes in this review. A recent application of NHSN criteria in pediatric hospitals in the United States demonstrated that MBI accounted for 40% of CLABSI in immunocompromised pediatric patients, which replicates the findings in our study (11). While strategies to decrease mucositis, such as oral rinses, cryotherapy, and low level light therapy have been recommended in high resource settings, this will not prevent all MBI (12). Moreover, the feasibility of implementation and the cost vs. benefit of interventions will have to be evaluated within the local context. The findings quantify the high risk of infectious complications of cancer-directed therapy in settings with limited resources and suggest that infection prevention and management must be prioritized to improve cancer outcomes.

Detailed investigation of antimicrobial susceptibility and time to effective therapy is required to pinpoint drivers of poor outcomes. Studies in adult patients with cancer have shown that delayed antimicrobial therapy may be associated with higher mortality due to resistant Gram-negative bacteria (13). During the time of study, antibiotic coverage in patients presenting with febrile neutropenia targeted Gram-negative organisms. Empiric coverage combined amikacin with either piperacillin-tazobactam or cefepime or meropenem based on the severity of the patient's clinical presentation. The predominance of Gram-negative bacteria and documented high usage of carbapenems, aminoglycosides, colistin, and polymyxin B at this center suggest high levels of antibiotic resistance. The study period included an outbreak of multidrug-resistant *Acinetobacter baumanii* between January 2013 and June 2015 which resulted in high mortality (14). In our study, 64% of *Acinetobacter baumanii* infections resulted in BSI-related mortality; however, the role of inappropriate or delayed antimicrobial therapy is not assessable from our data.

Frequent *Klebsiella pneumoniae* isolation supports the hypothesis that antibiotic resistance may have affected outcomes. The organism has a wide range of virulence properties and antimicrobial-resistance mechanisms, some of which can be acquired by other Gram-negative bacteria *via* horizontal gene transmission (15). While the pediatric oncology population is not well-characterized, data from adult patients suggest that plasmid-encoded carbapenemases circulate in this geographic area (16). Infections by carbapenem-resistant Enterobacteriaceae are associated with high mortality in adults (17). Although an Australian study in pediatric oncology patients found no mortality differences between patients with susceptible and resistant Gram-negative bacteria, increased rates of clinical complications and longer hospitalizations occurred in the antibiotic-resistant infection group (18). We found that Gram-negative isolates were not significantly associated with increased mortality or ICU admission; however, correlation with susceptibility results and molecular determinants of resistance may identify high-risk subgroups.

The study's strengths include the extended review period and targeted dataset. Data collection challenges, however, led to a number of limitations. First, the study was limited in its ability to review antimicrobial treatment and prophylaxis. As a result we could not assess effectiveness of initial antibiotic therapy against the isolated organism. Second, we did not have susceptibility data; however, the observed morbidity and mortality may reflect the outbreak of MDR *Acinetobacter baumaunii* that occurred during the study period. Third, we could not compare outcomes of patients with BSI to patients without BSI. Fourth, we may have underestimated BSI due to commensal bacteria: In a healthy host, this underestimation may be clinically irrelevant; however, these organisms can cause severe disease in immunocompromised patients. It is unclear whether including these organisms would have changed the observed morbidity and mortality. In particular exclusion of single isolates of coagulase negative staphylococci may have skewed the distribution of our isolates toward Gram negative bacilli relative to other studies (19, 20). Nevertheless, as only 8% of episodes in our study occurred in patients with a permanent central venous line, these organisms were more likely to be contaminants than in cohorts where permanent venous line use was more prevalent.

In general, our limitations underscore the importance of accurately and consistently capturing laboratory results that are linked to clinical data. It is a call to action for institutions to prioritize funding and personnel for data management since improvement in treatment outcomes cannot be measured or achieved without data (21). Since the time of this study, UNOP has improved its infrastructure and personnel for laboratory and clinical data collection. Infectious diseases consultants were available and recommended a consistent approach to fever management throughout the study period. Nevertheless, the standard algorithm for fever management, including diagnostic and treatment recommendations, was approved afterwards. Studies of provider adherence to the treatment algorithm and outcomes of algorithm implementation are ongoing. These studies will also examine the association of susceptibility patterns with clinical outcomes. Finally, further work is needed to explore the acceptability, feasibility and effectiveness of infection prevention, including catheter-management bundles and mucositis prevention, in this setting (22).

In conclusion, our study highlights that optimizing infection diagnosis and management is pivotal to improving global childhood cancer outcomes. The disproportionately high BSI-related morbidity and mortality we observed in this population is likely multifactorial and will require a multimodal response addressing aspects of prevention, diagnosis, and treatment. Our work highlights the need for further studies on infection prevention and treatment specific to this setting. With greater investment in data support, locally applicable evidence will be generated, and patient outcomes can be improved.

## Data availability statement

The original contributions presented in the study are included in the article/supplementary material, further inquiries can be directed to the corresponding authors.

## Ethics statement

The studies involving human participants were reviewed and approved by Universidad Francisco Marroquin Ethics Committee and St. Jude Children's Research Hospital's Institutional Review Board. Written informed consent from the participants' legal guardian/next of kin was not required to participate in this study in accordance with the national legislation and the institutional requirements.

## Author contributions

SM conceptualized the study, wrote the manuscript, and approved the final manuscript as submitted. MM and AC conceptualized the study, supervised data collection, revised the manuscript, and approved the final manuscript as submitted. CB completed data collection and entry, revised the manuscript, and approved the final manuscript as submitted. MH and MG developed and revised the data collection instruments and database, reviewed the data, revised the manuscript, and approved the final manuscript as submitted. FA conceptualized the study, revised the manuscript, and approved the final manuscript as submitted. YS and LT provided statistical analysis for the study, revised the manuscript, and approved the final manuscript as submitted. MC conceptualized the study, supervised data collection, critically reviewed the manuscript, and approved the final manuscript as submitted. All authors contributed to the article and approved the submitted version.

## Funding

This work was supported by a Pediatric Infectious Diseases Society of America–St. Jude Fellowship Award in Basic and Translational Research (to SM) and by ALSAC.

## Conflict of interest

MM has an unrestricted research grant and received honorariums as a consultant from Pfizer vaccines. The remaining authors declare that the research was conducted in the absence of any commercial or financial relationships that could be construed as a potential conflict of interest.

## Publisher's note

All claims expressed in this article are solely those of the authors and do not necessarily represent those of their affiliated organizations, or those of the publisher, the editors and the reviewers. Any product that may be evaluated in this article, or claim that may be made by its manufacturer, is not guaranteed or endorsed by the publisher.

## Abbreviations

ANC: absolute neutrophil count
BSI: bloodstream infection
CLABSI: central line associated bloodstream infection
HAI: healthcare associated infection
ICU: intensive care unit
LCBI: laboratory confirmed bloodstream infection
MBI: Mucosal barrier injury
NHSN: National Healthcare Safety Network
UNOP: Unidad Nacional de Oncologia Pediatrica.
